# Educators’ Satisfaction with the Wellness4Teachers, a Psychological Intervention Program

**DOI:** 10.1192/j.eurpsy.2026.11361

**Published:** 2026-07-31

**Authors:** B. Agyapong, P. Brett-MacLean, Y. Wei, V. I. O. Agyapong

**Affiliations:** 1 Psychiatry, University of Alberta, Edmonton; 2 Psychiatry, Dalhousie University, Halifax, Canada

## Abstract

**Introduction:**

Educators’ psychological well-being is important as it indirectly influences students’ academic achievement and mental well-being. The Wellness4Teachers program, a daily supportive text messaging program, was launched in Canada to mitigate psychological problems among teachers.

**Objectives:**

This study assessed subscribers’ experiences and satisfaction with the Wellness4Teachers supportive text messaging program.

**Methods:**

This cross-sectional study was conducted during the 2022/2023 academic year and employed a mixed-methods approach. Data was collected from educators who self-subscribed to the free Wellness4Teachers program in Canada. Participants’ satisfaction levels were assessed using a Likert scale to evaluate the responses to different aspects of the program. Frequency counts and percentages were used to summarize quantitative responses. Qualitative data from responses to open-ended questions in the satisfaction survey were analyzed using content analysis.

**Results:**

Overall, 88.3% of the participants were female, and 11.7% were male. Overall, 68.2% of participants agreed that Wellness4Teachers text messages helped them manage stress, 63.1% agreed it helped them cope with anxiety, and 74.4% felt connected to a support system. Additionally, 73.6% reported improved overall mental well-being due to the program, and 72.2% felt hopeful about managing concerns with their mental health. Further, 95.4% of respondents reported that the text messages were always or often positive, and 93.3% deemed them to be always or often affirming. Qualitative feedback highlighted preferences for message delivery times and suggestions for enhancing personalization features to improve the Wellness4Teachers program.

**Conclusions:**

This study demonstrates that educators were highly satisfied with and receptive to the Wellness4Teachers program. With the addition of enhanced personalization features suggested by the educators, the program may help to effectively supplement support for teachers’ psychological well-being while offsetting challenges associated with time constraints and geographical location.

**Disclosure of Interest:**

None Declared

